# Visceral adiposity index score indicated the severity of coronary heart disease in Chinese adults

**DOI:** 10.1186/1758-5996-6-143

**Published:** 2014-12-22

**Authors:** Lu Han, Kai-li Fu, Jing Zhao, Zhi-hao Wang, Meng-xiong Tang, Jia Wang, Hui Wang, Yun Zhang, Wei Zhang, Ming Zhong

**Affiliations:** Key Laboratory of Cardiovascular Remodeling and Function Research, Chinese Ministry of Education and Chinese Ministry of Public Health, Department of Cardiology, Qilu Hospital of Shandong University, No.107, Wen Hua Xi Road, Ji’nan, 250012 P.R. China; Department of Geriatrics Medicine, Qilu Hospital of Shandong University, Ji’nan, 250012 P.R. China; Department of Emergency Medicine, Qilu Hospital of Shandong University, Ji’nan, 250012 P.R. China

**Keywords:** Visceral adiposity index, Coronary heart disease, Diabetes

## Abstract

**Background:**

Visceral adiposity contributes to cardiometabolic risk, and visceral adiposity index (VAI) had significant correlation with visceral adiposity. We aimed to explore whether VAI was associated with cardiac structure and function and assess the impact of the cut-off points of VAI defining visceral adipose dysfunction (VAD) on the severity of coronary heart disease (CHD).

**Methods:**

A total of 95 patients with CHD were divided into Control (nondiabetic CHD patients) and DM group (diabetic CHD patients). Then the two groups were respectively divided into VAD absent and VAD groups. Clinical, echocardiographic and coronary artery angiographic indexes were acquired to examine in relation to VAI.

**Results:**

A significant increasing trend among the four groups of patients (Control + VAD absent, Control +VAD, DM + VAD absent and DM +VAD groups) were observed for waist circumference (WC), body mass index (BMI), systolic blood pressure (SBP), glucose, VAI and Gensini score (*P*<0.05 for all). The following variables were associated with VAI: total cholesterol, nonesterified fatty acid, Waist-Hip ratio and SBP. VAI was independently associated with Gensini score.

**Conclusions:**

The extent of CHD was more severe in diabetes, and VAI as a simple indicator of visceral adipose mass was strongly associated with the severity of CHD. The cut-off points of VAI used for defining VAD were more useful in diabetic CHD patients in identifying the severity of CHD.

## Background

The increased prevalence of obesity contributes to cardiovascular disease (CVD) risk [1]. However, not every obese patient develops CVD. In this regard, visceral adiposity has been found to play a key role in cardiometabolic risk [2]. The precise measurement of the total amount of body fat and its regional distribution is possible by using computed tomography (CT) or Magnetic resonance imaging (MRI) [3], but they are costly and not routinely available. Accordingly there is a need for simple techniques that can identify visceral adiposity, such as waist circumference (WC), but WC alone does not help in distinguishing between subcutaneous and visceral (both omental and mesenteric) fat mass [4]. Amato et al [5] has recently developed a novel sex-specific index based on WC, body mass index (BMI), triglyceride (TG), and high-density lipoprotein (HDL) cholesterol and termed it visceral adiposity index (VAI), and observed that VAI had significant correlation with visceral adiposity. And VAI showed a strong independent association with cardiovascular risk [5]. Clinical studies have indicated that visceral adipose tissue was associated with cardiac structure and function [6]. However, the correlations between VAI and cardiac structure and function as well as that between VAI and the severity of coronary heart disease (CHD) remain unclear.

Diabetes is a major risk factor of incident CVD and VAI showed higher correlation with incident diabetes events than its individual components (WC, BMI, TG and HDL) [7]. However, the contribution of diabetes to the severity of CHD remains unclear. Recently, Amato et al identified in a Caucasian Sicilian population the age-stratified cut-off points of VAI, patients with VAI scores greater than these cut-off points were arbitrarily defined as visceral adipose dysfunction (VAD) [8]. To better understand the correlations between VAI and cardiac structure and function and the impact of the cut-off points of VAI defining VAD on the severity of CHD, we used simple anthropometric measures, echocardiography and coronary angiography and examined: first, whether diabetes and VAD aggravated the severity of coronary artery disease of CHD patients. Second, whether there were other clinical influences of VAI. Finally, we determined the correlations between VAI and cardiac structure and function.

## Methods

### Study population

We recruited 95 CHD patients diagnosed by coronary angiography on admission to Qilu Hospital of Shandong University with the complaints suggestive of angina or myocardial infarction. All subjects were unrelated Chinese Han nationality. Then, the selected CHD patients were divided into two main groups: Control group (nondiabetic CHD patients) and DM group (diabetic CHD patients). Informed consent was obtained from all subjects based on a protocol approved by the Ethics Committee of Qilu Hospital of Shandong University.

### Clinical assessment

The details of age, gender, weight and height were obtained, with BMI calculated as the body weight in kilograms divided by the height in meters squared. WC was measured at the level of the umbilicus, systolic and diastolic blood pressures (SBP and DBP) were obtained with a mercury sphygmomanometer using auscultatory methods. Blood was drawn after the participants had fasted overnight. Levels of plasma total cholesterol (TC), HDL, low-density lipoprotein (LDL) cholesterol, TG, nonesterified fatty acid (NEFA) and fasting blood glucose were measured by standard laboratory techniques.

VAI score was calculated as described [5] using the following sex-specific equations, when TG is Triglycerides levels expressed in mmol/l and HDL is HDL-cholesterol levels expressed in mmol/l:


### Echocardiography

Echocardiograms were obtained with a commercially available ultrasound machine (Vivid 7; GE Vingemed Ultrasound, Horten, Norway) with a 2.5 or 3.5 MHZ phased array transducer. Participants were examined in the left lateral decubitus position and images were acquired at passive end expiration to minimise global cardiac movement from standard parasternal long axis and apical planes.

The M-mode echocardiographic study of the left ventricles performed under 2D control. The ventricular septal (VS) and posterior wall (PW) thicknesses at end-diastole, and left ventricular end-diastolic dimensions (LVDd) were determined from M-mode echocardiogram by American Society of Echocardiography recommendations. The left ventricular ejection fraction (LVEF) was estimated by the modified Simpson method and was used as the parameter of left ventricular (LV) systolic function.

### Gensini score

To assess the severity of CHD, we used the Gensini scoring system [9]. Coronary artery score equals the sum of all segment scores (where each segment score equals segment weighting factor multiplied by severity score). Severity scores assigned to the specific percentage luminal diameter reduction of the coronary artery segment are 32 for 100%, 16 for 99%, 8 for 90%, 4 for 75%, 2 for 50%, and 1 for 25%.

### Statistical analysis

Data are presented as mean ± SD for continuous variables and as proportions for categoric variables. One-way ANOVA was used to compare the differences among groups of participants. Stepwise multiple regression analysis was performed to determine the correlation and independent variables for VAI as well as Gensini score. Statistical analyses were performed using SPSS 17.0 (SPSS Inc., Chicago, IL) software. A two-tailed *P* value < 0.05 was considered statistically significant.

## Results

The demographic and clinical characteristics of the CHD participants are shown in Table 1. The subjects included 60 males and 35 females, and their mean age was 60.89 ± 8.66 years. There were no significant differences in age, gender, smoking, aspirin and stain treatments between Control group and DM group. DM group had significantly higher body weight, WC, Waist-Hip ratio, SBP, BMI, fasting glucose, TC, TG, NEFA and Gensini score and lower HDL which showed that diabetes mellius had more serious glucose metabolic disorders, dyslipidemia and coronary lesion.

**Table 1:** **Baseline characteristic of CHD patients with or without diabetes**

	Control (n = 57)	DM (n =38)	***P***
Age (years)	60.09 ± 9.10	61.68 ± 8.45	0.391
Gender (M/F)	38/19	22/16	0.396
Body weight (kg)	66.03 ± 7.97	70.21 ± 7.87	0.014
WC (cm)	91.00 ± 6.67	97.18 ± 7.64	0.000
Waist-Hip ratio	0.95 ± 0.04	0.97 ± 0.06	0.015
BMI (kg/m2)	24.72 ± 2.63	26.13 ± 2.59	0.012
SBP (mmHg)	130.81 ± 16.32	142.05 ± 17.12	0.002
DBP (mmHg)	74.68 ± 7.93	74.55 ± 12.00	0.953
Glucose (mmol/l)	4.91 ± 0.64	7.35 ± 1.93	0.000
TC (mmol/l)	4.33 ± 0.94	4.77 ± 1.12	0.041
TG (mmol/l)	1.29 ± 0.64	1.59 ± 0.81	0.046
NEFA (mmol/l)	86.09 ± 51.86	115.95 ± 64.40	0.020
HDL (mmol/l)	1.19 ± 0.24	1.08 ± 0.22	0.035
LDL (mmol/l)	2.78 ± 0.77	2.83 ± 0.82	0.774
Gensini	63.47 ± 48.44	106.66 ± 32.91	0.000
VAI	1.80 ± 1.08	2.61 ± 1.59	0.008
Smoking	17 (0.30)	16 (0.42)	0.273
Hypertension	36 (0.63)	26 (0.68)	0.664
β-blocker	13 (0.23)	12 (0.32)	0.353
ACEI	7 (0.12)	5 (0.13)	1.000
ARB	6 (0.11)	4 (0.11)	1.000
Statin	11 (0.19)	7 (0.18)	1.000
Aspirin	26 (0.46)	18 (0.47)	1.000

Data are mean ± SD or n (proportion).

CHD: coronary heart disease; DM: diabetes mellitus; VAD: visceral adipose dysfunction; WC: Waist circumference; BMI: body mass index; SBP: systolic blood pressures; DBP: diastolic blood pressures; TC: Total cholesterol; TG: triglyceride; NEFA: nonesterified fatty acid; HDL: high-density lipoprotein cholesterol; LDL: low-density lipoprotein cholesterol; VAI: visceral adiposity index; ACEI: angiotensin-converting enzyme inhibitor; ARB: angiotensin receptor blocker.

According to the study of Amato et al [8], the appropriate cut-off points of VAI for defining VAD were 2.52 for patients under 30 years, 2.23 for those aged between 30 and 42 years, 1.92 between 42 and 52 years, 1.93 between 52 and 66 years and 2.00 for patients over 66 years. We divided Control group into Control + VAD absent group and Control +VAD group, and divided DM group into DM + VAD absent group and DM + VAD group. The baseline characteristics of the four groups are shown in Table 2. A significant increasing trend among the four groups of patients (Control + VAD absent group, Control +VAD group, DM + VAD absent group and DM + VAD group ) were observed for body weight (*P* = 0.010), WC (*P* < 0.001), Waist-Hip ratio (*P* = 0.024), BMI (*P* = 0.011), SBP (*P* = 0.020), Glucose (*P* < 0.001), NEFA (*P* = 0.044) and VAI (*P* = 0.008). A significant increasing trend among the four groups of patients were also observed for Gensini score (*P* = 0.001), but VAD and DM showed no significant interaction (*P*=0.883). There were also significant differences in TG (*P* = 0.002) and HDL (*P* = 0.0016) among the four groups.

**Table 2:** **Baseline characteristic of Control + VAD absent, Control + VAD, DM + VAD absent and Control + VAD patients**

	Control + VAD absent (n = 35)	Control + VAD (n = 22)	DM + VAD absent (n =17)	DM + VAD (n =21)	***P***
Age (years)	59.71 ± 9.09	60.68 ± 9.30	63.47 ± 9.59	60.24 ± 7.32	0.546
Gender (M/F)	25/10	13/9	13/4	9/12	0.102
Body weight (kg)	64.26 ± 6.80	68.86 ± 6.01*	69.09 ± 7.19*	71.12 ± 8.44*	0.010
WC (cm)	89.71 ± 5.95	93.05 ± 7.33	94.29 ± 7.86*	99.52 ± 6.75*^†#^	0.000
Waist-Hip ratio	0.95 ± 0.04	0.95 ± 0.04	0.96 ± 0.05	0.99 ± 0.06*^†^	0.024
BMI (kg/m2)	24.49 ± 2.96	25.09 ± 2.00	25.18 ± 1.65	26.89 ± 2.98*^†#^	0.011
SBP (mmHg)	130.2 ± 14.31	131.8 ± 19.43	141.6 ± 19.30*	142.4 ± 15.64*^†^	0.020
DBP (mmHg)	73.51 ± 7.30	76.55 ± 8.70	72.47 ± 13.00	76.24 ± 11.15	0.439
Glucose (mmol/l)	4.87 ± 0.65	4.96 ± 0.64	7.19 ± 1.70*^†^	7.47 ± 2.13*^†^	0.000
TC (mmol/l)	4.36 ± 0.94	4.29 ± 0.95	4.46 ± 0.95	5.03 ± 1.21	0.067
TG (mmol/l)	1.27 ± 0.61	1.32 ± 0.71	1.17 ± 0.62	1.93 ± 0.19*^†#^	0.002
NEFA (mmol/l)	86.14 ± 50.51	86.00 ± 55.14	100.65 ± 60.90	128.3 ± 65.92*^†^	0.044
HDL (mmol/l)	1.23 ± 0.25	1.11 ± 0.20	1.176 ± 0.28	1.01 ± 0.12*^#^	0.006
LDL (mmol/l)	2.78 ± 0.79	2.79 ± 0.76	2.58 ± 0.62	3.03 ± 0.91	0.359
Gensini	52.66 ± 39.27	80.68 ± 30.63*	93.76 ± 20.07*	117.1 ± 37.76*^†#^	0.001
Smoking	12 (0.34)	5 (0.23)	12 (0.71)	4 (0.19)	0.004
Hypertension	24 (0.69)	12 (0.55)	12 (0.71)	14 (0.67)	0.679
β-blocker	9 (0.26)	4 (0.18)	4 (0.24)	8 (0.38)	0.507
ACEI	6 (0.17)	1 (0.06)	5 (0.29)	0 (0.00)	0.025
ARB	5 (0.14)	1 (0.06)	1 (0.06)	3 (0.14)	0.559
Statin	9 (0.26)	2 (0.09)	2 (0.12)	5 (0.24)	0.344
Aspirin	18 (0.51)	8 (0.36)	10 (0.59)	8 (0.38)	0.410

Data are mean ± SD or n (proportion).

VAD: visceral adipose dysfunction; DM: diabetes mellitus; WC: Waist circumference; BMI: Body mass index; SBP: systolic blood pressures; DBP: diastolic blood pressures; TC: Total cholesterol; TG: triglyceride; NEFA: nonesterified fatty acid; HDL: high-density lipoprotein cholesterol; LDL: low-density lipoprotein cholesterol; ACEI: angiotensin-converting enzyme inhibitor; ARB: angiotensin receptor blocker. *P < 0.05 vs Control + VAD absent; ^†^ *P <* 0.05 vs Control+ VAD; ^#^ *P <* 0.05 vs DM+ VAD absent.

After the adjustment for the effect of age, gender and the four clinical indexes in the mathematical model of VAI, the multivariate models including SBP, DBP, heart rate, Waist-Hip ratio, Glucose, TC, LDL and NEFA factors. The following variables were associated with VAI: TC, NEFA, Waist-Hip ratio and SBP (Table 3).

**Table 3:** **Multiple linear regression for relation of VAI to clinical factors**

	Beta	R square	***P***
TC (mmol/l)	0.395	0.148	<0.001
NEFA (μmol/dl)	0.313	0.200	0.001
Waist-Hip ratio	0.236	0.244	0.012
SBP (mmHg)	0.208	0.284	0.027

WC: Waist circumference; BMI: Body mass index; TG: triglyceride; TC: Total cholesterol; NEFA: nonesterified fatty acid; SBP: systolic blood pressures. Beta: Standardized Coefficients.

After the adjustment for the effect of age, gender, the multivariate models including the ultrasonic parameters left atrial (LA) diameter, LV diameter, right ventricular (RV) diameter, aortic (AO) diameter, pulmonary artery (PA) diameter, LVEF and left atrial dimension index (LADI). The following variables were associated with VAI: LADI and PA (Table 4).

**Table 4:** **Multiple linear regression for relation of VAI to ultrasonic index**

	Beta	R square	***P***
LADI (mm/m^2^)	0.323	0.092	0.020
PA (mm)	0.299	0.181	0.030

VAI: visceral adiposity index; LADI: 1eft atrial dimension index; PA: pulmonary artery; Beta: Standardized Coefficients.

Stepwise multiple linear regression analysis was also used to determine independent variables of Gensini score. The model included age, SBP, DBP, Glucose, TC, LDL and VAI as independent variables and the analysis revealed that VAI (OR 18.257 [95%CI 6.038-30.475]; *P* = 0.005) was independently associated with Gensini score.

## Discussion

The study compared clinical indexes, cardiac structure and function and the severity of coronary artery diseases between nondiabetic CHD patients and diabetic CHD patients. The major findings of the present study lead us to the following conclusions: [1] The CHD patients without diabetes had no significant differences in glucose and lipid metabolism disorders between VAD absent group and VAD group, but the extent of CHD of VAD group was more severe than VAD absent group. However, VAD aggravated glucose and lipid metabolism disorder and the severity of coronary artery diseases of CHD patients with diabetes. [2] After adjusting for age and sex, TC, NEFA, Waist-Hip ratio and SBP contributed to VAI; [3] VAI had independent association with Gensini score.

Diabetes is associated with an increased risk of cardiovascular death and a higher incidence of CHD [10]. One of the features of diabetes is abdominal obesity, and visceral adipose tissue plays an importance role in the progress of diabetes. VAI was strongly associated with incident diabetes. In this study, we found that DM + VAD group had significant increases in glucose and lipid metabolism disorders and the severity of CHD, but the classification of VAD according to the aged cut-off points of VAI had no obvious advantage in CHD patients without diabetes. Consequently, VAD which Amato et al. identified played a more importance role in CHD patients with diabetes.

Smoking is associated with the development of CHD and influences short and long term outcomes of patients who smoke after diagnosis [11, 12]. There is increasing evidence that smoking affects body fat distribution and that it is associated with central obesity [13]. Therefore, smoking might contribute to VAI and be a risk factor for VAD, thus aggravating the severity of CHD. Drug therapy also influences the severity of CHD. Statin treatment ameliorates the severity of CHD through the control of plasma cholesterol level. Furthermore, it has been demonstrated that statin treatment reduced adiposity of *ob/ob* mice [14]. Aspirin is also a kind of drug which can protect CHD through its antiplatelet effect [15]. In this study, there were no significant differences in smoking, statin and aspirin treatments between CHD without diabetes and diabetic CHD. Therefore, the effects of smoking, statin and aspirin treatments are equal between CHD without diabetes and diabetic CHD.

Previous studies have investigated that VAT/SAT (subcutaneous adipose tissue) was associated with higher SBP, DBP, lower HDL-cholesterol and higher TG [16]. In the study, we found TC, NEFA, Waist-Hip ratio and SBP could also contribute to VAI. Visceral adipose tissue was independently associated with left ventricular mass and LA enlargement [6, 17], and low levels of adiponectin released from adipose tissue are directly linked to the development of pulmonary arterial hypertension [18]. Our results suggested VAI was positively correlated with LA and pulmonary artery enlargement and was independently associated with the severity of CHD.

### Limitations

In our study, we defined VAD through the cut-off points derived from a Caucasian Sicilian population that Amato et al. study and found that VAD aggravated the severity of CHD in Chinese adults, suggesting that the cut-off points of VAI could be applied to CHD of Chinese in some extent. However, the exact cut-off points of VAI suitable for CHD of Chinese adult patients should be determined.

## Conclusions

In conclusion, the extent of CHD was more severe in diabetes, and VAI as a simple indicator of visceral adipose mass was strongly associated with the severity of CHD. The cut-off points of VAI which Amato et al used for defining VAD were more useful in CHD patients with diabetes in identifying the severity of CHD.

## Abbreviations

VAI: Visceral adiposity index
VAD: Visceral adipose dysfunction
CHD: Coronary heart disease
CVD: Cardiovascular disease
CT: Computed tomography
MRI: Magnetic resonance imaging
WC: Waist circumference
BMI: Body mass index
SBP: Systolic blood pressures
DBP: Diastolic blood pressures
NEFA: Nonesterified fatty acid
TC: Total cholesterol
TG: Triglyceride
LDL: Low-density lipoprotein cholesterol
HDL: High-density lipoprotein cholesterol
LADI: Left atrial dimension index
PA: Pulmonary artery
VS: Ventricular septal
PW: Posterior wall
LVDd: Left ventricular end-diastolic dimensions
LVEF: Left ventricular ejection fraction
LA: Left atrial
RV: Right ventricular
AO: Aortic
PA: Pulmonary artery.

## References

[1] Bozorgmanesh MR, Hadaegh F, Padyab M, Mehrabi Y, Azizi F (2008). Temporal changes in anthropometric parameters and lipid profile according to body mass index among an adult Iranian urban population. Ann Nutr Metab.

[2] Després JP (2006). Intra-abdominal obesity: an untreated risk factor for Type 2 diabetes and cardiovascular disease. J Endocrinol Investig.

[3] Flegal KM, Shepherd JA, Looker AC, Graubard BI, Borrud LG, Ogden CL, Harris TB, Everhart JE, Schenker N (2009). Comparisons of percentage body fat, body mass index, waist circumference, and waist-stature ratio in adults. Am J Clin Nutr.

[4] Pouliot MC, Despres JP, Lemieux S, Moorjani S, Bouchard C, Tremblay A, Nadeau A, Lupien PJ (1994). Waist circumference and abdominal saggitaldiameter: best simple anthropometric indices of abdominal visceral adipose tissue accumulation and related cardiovascular risk in men and women. Am J Cardiol.

[5] Amato MC, Giordano C, Galia M, Criscimanna A, Vitabile S, Midiri M, Galluzzo A, AlkaMeSy Study Group (2010). Visceral Adiposity Index: A reliable indicator of visceral fat function associated with cardiometabolic risk. Diabetes Care.

[6] Liu J, Fox CS, Hickson DA, May WL, Ding J, Carr JJ, Taylor HA (2011). Pericardial fat and echocardiographic measures of cardiac abnormalities: the Jackson Heart Study. Diatetes Care.

[7] Bozorgmanesh M, Hadaegh F, Azizi F (2011). Predictive performance of the visceral adiposity index for a visceral adiposity-related risk: type 2 diabetes. Lipids Health Dis.

[8] Amato MC, Giordano C, Pitrone M, Galluzzo A (2011). Cut-off points of the visceral adiposity index (VAI) identifying a visceral adipose dysfunction associated with cardiometabolic risk in a Caucasian Sicilian population. Lipids Health Dis.

[9] Gensini GG (1983). A more meaningful scoring system for determining the severity of coronary heart disease. Am J Cardiol.

[10] Poulsen MK, Henriksen JE, Dahl J, Johansen A, Gerke O, Vach W, Haghfelt T, Høilund-Carlsen PF, Beck-Nielsen H, Møller JE (2010). Left ventricular diastolic function in type 2 diabetes mellitus prevalence and association with myocardial and vascular disease. Circ: Cardiovasc Imaging.

[11] van Domburg RT, op Reimer WS, Hoeks SE, Kappetein AP, Bogers AJ (2008). Three life-years gained from smoking cessation after coronary artery bypass surgery: a 30-year follow-up study. Am Heart J.

[12] Ashby DT, Dangas G, Mehran R, Lansky AJ, Fahy MP, Iakovou I, Satler LF, Pichard AD, Kent KM, Stone GW, Leon MB (2002). Comparison of one-year outcomes after percutaneous coronary intervention among current smokers, ex-smokers, and nonsmokers. Am J Cardiol.

[13] Chiolero A, Faeh D, Paccaud F, Cornuz J (2008). Consequences of smoking for body weight, body fat distribution, and insulin resistance. Am J Clin Nutr.

[14] Khan T, Hamilton MP, Mundy DI, Chua SC, Scherer PE (2009). Impact of simvastatin on adipose tissue: pleiotropic effects in vivo. Endocrinology.

[15] Grove EL (2012). Antiplatelet effect of aspirin in patients with coronary artery disease. Dan Med J.

[16] Kaess BM, Pedley A, Massaro JM, Murabito J, Hoffmann U, Fox CS (2012). The ratio of visceral to subcutaneous fat, a metric of body fat distribution, is a unique correlate of cardiometabolic risk. Diabetologia.

[17] Malavazos AE, Morricone L, Marocchi A, Ermetici F, Ambrosi B, Corsi MM (2006). N-terminal pro-B-type natriuretic peptide and echocardiographic abnormalities in severely obese patients: correlation with visceral fat. Clin Chem.

[18] Summer R, Walsh K, Medoff BD (2011). Obesity and pulmonary arterial hypertension: Is adiponectin the molecular link between these conditions?. Pulm Circ.

